# A mosquito mouthpart-like bionic neural probe

**DOI:** 10.1038/s41378-023-00565-5

**Published:** 2023-07-12

**Authors:** Yu Zhou, Huiran Yang, Xueying Wang, Heng Yang, Ke Sun, Zhitao Zhou, Liuyang Sun, Jianlong Zhao, Tiger H. Tao, Xiaoling Wei

**Affiliations:** 1 State Key Laboratory of Transducer Technology, Shanghai Institute of Microsystem and Information Technology, Chinese Academy of Sciences, 200050 Shanghai, China; 2 School of Graduate Study, University of Chinese Academy of Sciences, 100049 Beijing, China; 3 2020 X-Lab, Shanghai Institute of Microsystem and Information Technology, Chinese Academy of Sciences, 200050 Shanghai, China; 4 Center of Materials Science and Optoelectronics Engineering, University of Chinese Academy of Sciences, 100049 Beijing, China; 5 Center for Excellence in Brain Science and Intelligence Technology, Chinese Academy of Sciences, 200031 Shanghai, China; 6 Neuroxess Co., Ltd. (Jiangxi), 330029 Nanchang, Jiangxi China; 7 Guangdong Institute of Intelligence Science and Technology, Hengqin, 519031 Zhuhai, Guangdong China; 8 Tianqiao and Chrissy Chen Institute for Translational Research, Shanghai, China

**Keywords:** Electrical and electronic engineering, Biosensors

## Abstract

Advancements in microscale electrode technology have revolutionized the field of neuroscience and clinical applications by offering high temporal and spatial resolution of recording and stimulation. Flexible neural probes, with their mechanical compliance to brain tissue, have been shown to be superior to rigid devices in terms of stability and longevity in chronic recordings. Shuttle devices are commonly used to assist flexible probe implantation; however, the protective membrane of the brain still makes penetration difficult. Hidden damage to brain vessels during implantation is a significant risk. Inspired by the anatomy of the mosquito mouthparts, we present a biomimetic neuroprobe system that integrates high-sensitivity sensors with a high-fidelity multichannel flexible electrode array. This customizable system achieves distributed and minimally invasive implantation across brain regions. Most importantly, the system’s nonvisual monitoring capability provides an early warning detection for intracranial soft tissues, such as vessels, reducing the potential for injury during implantation. The neural probe system demonstrates exceptional sensitivity and adaptability to environmental stimuli, as well as outstanding performance in postoperative and chronic recordings. These findings suggest that our biomimetic neural-probe device offers promising potential for future applications in neuroscience and brain-machine interfaces.

**Figure:**
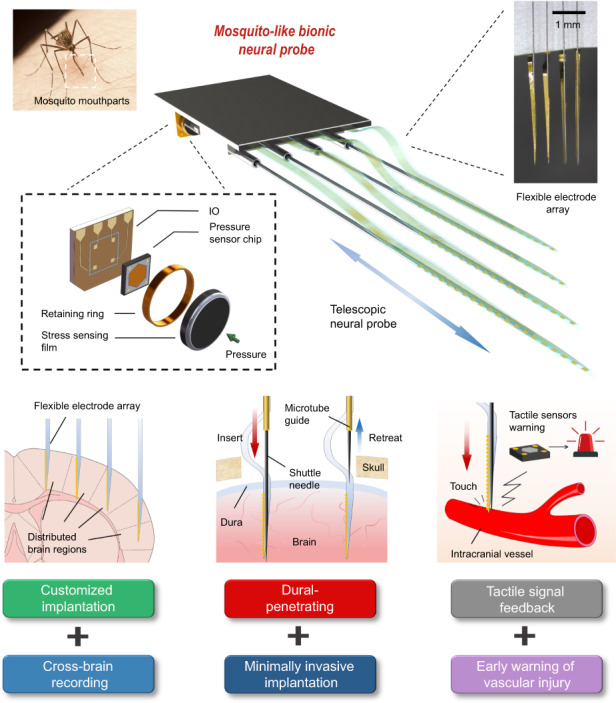
A mosquito mouthpart-like bionic neural probe consisting of a highly sensitive tactile sensor module, a flexible microelectrode array, and implanted modules that mimic the structure of mosquito mouthparts. The system enables distributed implantation of electrode arrays across multiple brain regions while making the implantation minimally invasive and avoiding additional dural removal. The tactile sensor array can monitor the implantation process to achieve early warning of vascular damage. The excellent postoperative short-term recording performance and long-term neural activity tracking ability demonstrate that the system is a promising tool in the field of brain-computer interfaces.

A mosquito mouthpart-like bionic neural probe consisting of a highly sensitive tactile sensor module, a flexible microelectrode array, and implanted modules that mimic the structure of mosquito mouthparts. The system enables distributed implantation of electrode arrays across multiple brain regions while making the implantation minimally invasive and avoiding additional dural removal. The tactile sensor array can monitor the implantation process to achieve early warning of vascular damage. The excellent postoperative short-term recording performance and long-term neural activity tracking ability demonstrate that the system is a promising tool in the field of brain-computer interfaces.

## Introduction

The use of invasive electrode arrays with high density and resolution offers significant potential for both basic neuroscience research and the clinical treatment of brain disorders such as epilepsy and amyotrophic lateral sclerosis (ALS)^1–4^. However, conventional invasive electrode arrays, which typically comprise silicon and metal, are mechanically incompatible with the intracranial tissue, leading to immune and inflammatory responses and shortening the device’s working lifespan^5–7^. To address these issues, flexible invasive devices comprising polymer materials, such as injectable mesh electronics^8,9^, nanoelectronic threads^10,11^, and flexible polymer fibers^12–14^, have been developed to mitigate the mechanical mismatch between the devices and the tissue. The excellent mechanical compliance of these devices results in reduced immune and inflammatory responses in the brain tissue, leading to more prominent chronic recording capabilities^6,15,16^. However, these devices lack the required stiffness to be implanted directly into brain tissue, such that using rigid shuttle devices to aid implantation is common practice. Nonetheless, the implantation process remains complex and risky due to several challenges: (1) The brain has surface topography includes curved surfaces and gullies, and the location of different brain regions are complexly mapped to the three-dimensional space, which makes it challenging to accurately implant electrodes into targeted brain regions. Existing shuttles often lack the necessary adjustable capacity to meet this challenge^17^. (2) The brain is protected by dense connective tissue such as the dura and arachnoid, making rigid implants prone to buckling during insertion. To overcome this, surgical removal of the dura mater before insertions is a common solution. However, dura removal can cause tissue damage, intraoperative infections, and swelling of brain tissue particularly in response to errors in depth targeting^18,19^. (3) Most importantly, complex vascular tissues are buried within the brain tissue, making it difficult to identify and avoid them during implantation through only visual inspection^20–22^. Simple and direct insertion of the probe increases the risk of damage to hidden blood vessels, leading to severe intracranial hemorrhage^23,24^. Despite some notable efforts, prior efforts have yet to address all three of these challenges simultaneously. Neuralink reported an implant device known as “Robert,” which can automatically implant individual flexible electrodes^25^. A complex optical system was equipped within it to identify shallow blood vessels to exclude potential vascular damage with the automatic execution of this device. The development of a neural probe system that is both freely customized and robust, as well as capable of real-time monitoring of the implanted environment, is highly desirable. Such a system would enable a minimally invasive and closed-loop implantation process.

As a classical bionics reference object, natural mosquito mouthparts offer several outstanding advantages in prototype implantation: (1) Flexible and adjustable scale to cope with complex skin surface topography^26^. (2) Excellent and minimally invasive penetration of the thicker epidermis^27,28^. (3) Acute sensory skills rather than vision to find and locate blood vessels^29^. Inspired by these bionics concepts, we designed a flexible and reliable neural probe system to overcome the common difficulties and risks of the implantation process. The neural probe device comprises a flexible electrode array, a multifunctional implant module with adjustable rigid shuttles, and a corresponding number of pressure sensors. Rigid shuttles equipped with sharpened tips allow for the precise and minimally invasive penetration of flexible electrode probes into brain tissues, avoiding the additional tissue damage and risk of infection associated with dura removal surgery^19^. The adjustable shuttle length is configurable to accommodate different brain surface morphologies, enabling implantation in various distributed brain regions. More importantly, the high-sensitivity sensor array can detect the stress of insertion and provide a means of distinguishing various intracranial tissues being contacted. In the event that the shuttle contacts a blood vessel, the abnormally high output amplitude of the sensor serves as an early warning mechanism, avoiding further damage. This feedback mechanism, akin to the positioning of vessels by mosquito mouthparts, eliminates the need for visual judgment and makes the implantation process more secure and efficient. Postimplantation, the implant module and sensor array can be separated and recycled, further enhancing the sustainability of the neural probe system. The high sensitivity sensor signal output, combined with its excellent postoperative and chronic recording performance, highlights the potential application of the neural probe system in the field of neuroscience and the treatment of brain diseases.

## Results

### Design and functionality of integrated sensing arrays

**Fig. 1:**
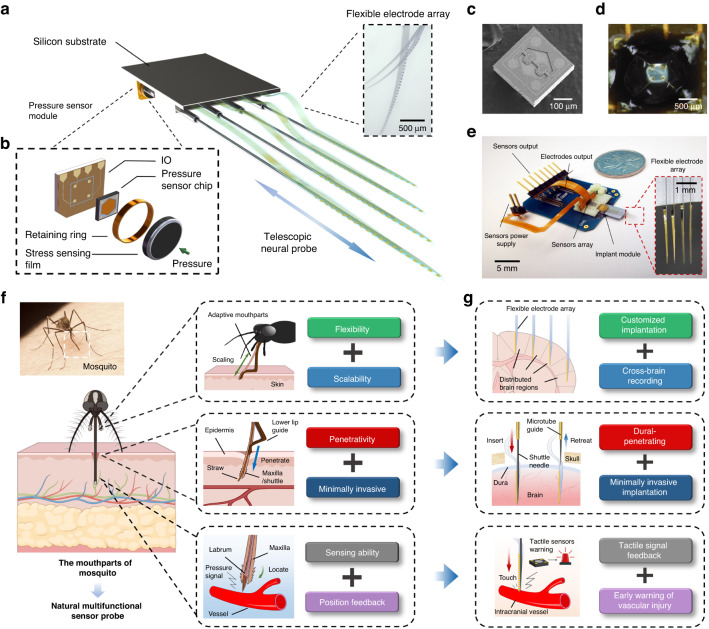
**Multifunctional biomimetic neural probe system, with multichannel flexible electrode array and high sensitivity sensor array**. **a** A schematic demonstrating the concept of a biomimetic neural probe system and the structure of the devices. **b** Schematic of the tactile sensor module structure. **c** SEM images of a tactile sensor chip. **d** Photo of the sensor integrated on the PCB board after wire bonding. **e** The assembled biomimetic neural probe system. A coin was used as a size comparison. The embedded picture shows the tip of a flexible electrode array mounted on the shuttles. **f** A diagram illustrating the blood-sucking process of a mosquito and the structure and function of its mouthparts. **g** Schematic illustration of the functions achieved through a biomimetic neural probe system, whose structure and function are references to the mosquito’s mouthparts

As new manufacturing techniques and materials are applied to develop minimally invasive electrodes, the size of neural probes has become further miniaturized^5,30,31^. However, effective methods to evaluate the interface mechanics between microelectrode probes and tissues are still lacking due to the deformations and tiny forces involved^32,33^. We expect to accurately distinguish and predict the various tissues contacted during implantation and to integrate and miniaturize the entire measurement system to avoid interference with surgery and reduce the case severity threshold for using implanted devices (Fig. 1a). To achieve this goal, we designed and manufactured a type of Wheatstone bridge-based tactile sensor with high sensitivity, high stability and a small footprint (0.42 × 0.42 mm, Fig. 1b, c). The sensors were transferred and integrated into the printed circuits, showing compliant attachment onto the substrate of the electrode probe via the movable and detachable 3D printing module (Fig. 1d). Ultrasonic wire bonding was used to lead out the I/O interface to meet the requirements of the compact arrangement of the device. Appropriate vinyl packaging was implemented as a protective measure to improve the stability and robustness of the device. The number of sensors in the array corresponds to the number of electrode probe shanks. The small size of the sensors and highly integrated package ensure that stress from each probe shank is accurately recorded. The test results of the output curve showed that the output voltage of the sensor has a stable linear relationship within the force range of ~10–100 kPa, and its sensitivity is 0.314 mV kPa^−1^, which is better than that of previous reports^34^. The high sensitivity and wide stable measurement range provided accurate sampling data for the subsequent analysis of interface forces with different tissues.

### Fabrication and electrical characterization of the flexible electrode array

Flexible electrodes integrated on a micron-thick polymer substrate provide a significant improvement in mechanical consistency with soft neural tissue compared to rigid electrodes, which is the premise for their superior chronic recording capability^8,35^. Moreover, compared with rigid electrodes, flexible electrodes can overcome the influence of the nonflat brain surface on the implantation accuracy and thus offer the potential to be freely configured in different target brain regions (however, this improvement depends on the accompanying implantation method)^36–38^. We fabricated a 128-channel ultraflexible electrode array using multilayer photolithography techniques as an invasive neural interface in conjunction with the probe system (Fig. 1a). Polyimide (PI) 2610 was selected as a flexible insulation layer of the device due to its excellent biocompatibility and flexibility. Each flexible electrode array comprised four free-standing shanks that were 2.5 μm thick, 105 μm wide on average, and 5 mm long. Thirty-two gold microelectrodes with a thickness of 100 nm were uniformly distributed in each shank (Fig. S1). The bending stiffness of the electrode array is ~4.23 × 10^−13^ N·m^2^, which is ~5 orders of magnitude lower than that of the commercial silicon probe (Fig. S6)^39^. The microelectrodes on the electrode array are electrically traced by the input/output (I/O) pads supported on the wafer substrate, and the electrical connection between the array and the amplifier module was manufactured by flip chip-bonded printed circuits. After, the electrode array was released from the carrier substrate by etching a prepatterned sacrificial nickel layer. The excess wafer substrate was subsequently removed, resulting in a free-standing flexible electrode array.

Reducing the interface impedance between the microelectrode and the surrounding liquid can effectively reduce thermal noise and improve the fidelity of neural signal recording. Conductive polymers such as poly(3,4-ethylenedioxythiophene) (PEDOT)^40,41^ and poly (pyrrole)^42^ are commonly used to modify microelectrode recording sites to reduce impedance and improve neural interfaces. Before assembling the flexible electrode array with the implant/sensing module, the microelectrodes were electrochemically modified with ethylenedioxythiophene (EDOT) and sodium dodecylbenzene sulfonate (SDBS). Impedance tests and scanning electron microscopy (SEM) were performed on the electroplated microelectrode. The PEDOT film with nanoscale roughness significantly improved the surface activity of the microelectrode while reducing the interface impedance by one order of magnitude (Fig. S7a, b), which is consistent with previous studies^43,44^. During the subsequent 16 weeks of implantation of the flexible electrode array in the mouse brain, the statistical impedance level remained at a consistently low level (Fig. S7c), demonstrating the long-term stability of this electrochemically modified neural interface.

### Design and manufacture of multifunctional adjustable implant modules

Silicon-based electrode probes based on micro/nanofabrication overcame the problem of channel density but lacked flexibility to match electrode stiffness to that of target brain regions^3,45^. Due to the radian and furrows on the surface of the brain and the complex spatial distribution of different brain regions, mechanical mismatch poses a new challenge to the working accuracy of invasive devices^46,47^. However, the shape of rigid probes and their distribution of recording sites cannot be adjusted specifically. Moreover, the protection of the brain from connective tissues such as the dura or arachnoid further challenge implantation of a probe (rigid or flexible)^18,19^. Implantation of the probe into brain tissue encounters similar challenges as mosquitoes face when hunting blood: uneven surface terrain (pores, body hair, and skin folds) and tough surface tissue (thicker epidermis)^29^. It is worth noting that mosquitoes have evolved flexible, multilayered, and multifunctional mouthparts to overcome these challenges during bloodsucking (Fig. 1f)^48^. The mosquito’s multilayer mouthparts can flexibly adjust their length to adapt to the epidermis of different thicknesses and the topography of the undulating skin. Meanwhile, the mosquito’s lower lip provides support and orientation for the invasive parts of the mouthparts (this structure increases the critical buckling force and avoids insertion force dissipation), and the sharp maxilla can easily penetrate the epidermis and guide the soft labrum to the blood vessels^48,49^.

**Fig. 2:**
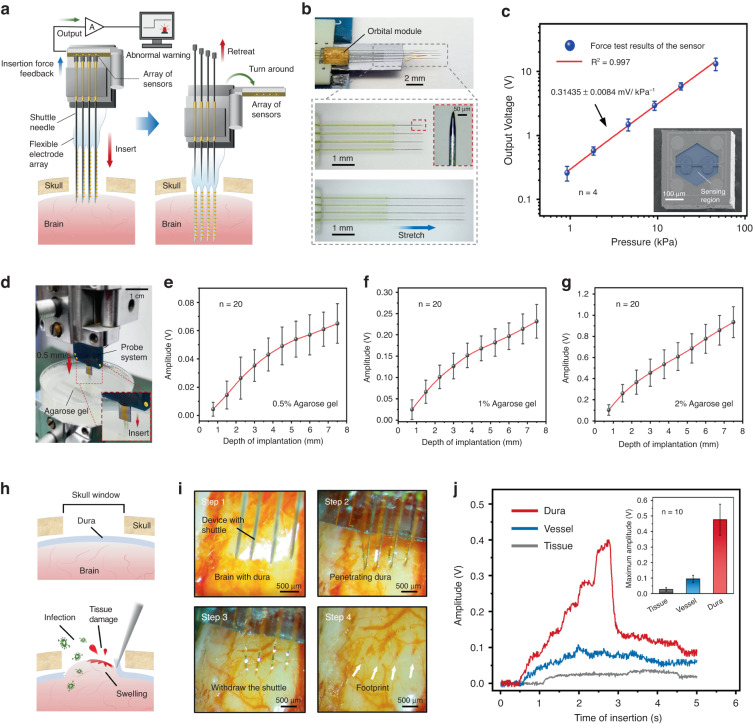
**Characterization of the sensor module and implantation test of the biomimetic neural probe system**. **a** A schematic to demonstrate how the neural probe system works in implantation. **b** Photos of the implant module and adjustable sharpened shuttles. **c** The sensitivity of the tactile sensors. **d** Photos of the in vitro implantation experiment and insertion force monitoring. Agarose with different mass fractions was used as the test object, and the test results are shown in **e**–**g**. The output signal is amplified by a 44 times amplifier. **h** A schematic of a dura removal procedure, which is required for the usual invasive probe implantation. This procedure is prone to additional intraoperative infections and injuries, as well as swelling of brain tissue. **i** Photographs show how the probe system penetrates the dura mater of a mouse and implants a flexible electrode array with 4 shanks. **j** The representative output voltage of the sensor module when the probe penetrates different tissues and the average peak value (embedded picture)

With reference to the above structures and principles, we designed a stable, adjustable, and highly penetrating implant module to help achieve minimally invasive distributed implantation of flexible electrode arrays (Fig. 1g). The implant module comprises several microtubule tracks with an internal diameter of 150 µm, rigid shuttles with directional mobility embedded in the tracks (the number of tracks and shuttles can be customized and expanded based on the number of electrode array shanks), and a base connecting the tracks, electrode substrate, and sensor array (Figs. 1e and 2a, b). Similar to the lower lip of the mosquito, the microtubule track provides support for the shuttles and stabilizes the orientation of the implant, concentrating the insertion force (Fig. 2b)^50^. Simultaneously, this avoids angular movement or buckling of the shuttle during implantation or retraction, avoiding potentially greater trauma. Each shuttle moves independently along the track, and its relative length can be adjusted or replaced according to different target brain regions, which can guide flexible probes to achieve highly customized distributed implantation. We used tungsten needles as shuttles to ensure a strong and penetrating interface, similar to the maxilla of the mosquito. The tungsten needle with an initial diameter of 75 µm was etched, and its front segment was refined to 40 µm and further sharpened into a vertebral body at the tip (Fig. 2b). The end of the shuttle is wrapped in a polyimide layer and flattened to constitute a contact surface with the sensor. Subsequent implant experiments demonstrated that these shuttles, which could be adjusted flexibly, could effectively reduce the insertion force required to penetrate protective tissues such as the dura. This indicates that implantation of the flexible electrode array will not require additional dura removal surgery^19^. The above design achievements provided a reliable guarantee for simplifying the operation process and reducing the risk of injury and intraoperative infection (Fig. 2h, i).

### In vitro implantation experiment and insertion force monitoring

We assembled the flexible electrode array, implant module and sensor array module into a complete probe system and conducted implantation test experiments. Insertion experiments were conducted on agarose phantom models (0.5%) that mechanically approximate cortical tissue, which were bathed in phosphate buffered saline (PBS) to mimic in vivo environments (Fig. 2d). The insertion force is continuously monitored through sensor output during the process of 1 mm/s constant insertion. As shown in Fig. 2e, the output of the sensor is positively correlated with the rate of penetration of the probe into the agarose phantom, with an approximate growth rate of 9 mV/mm and a corresponding insertion force growth rate of 0.07 mN/mm (the output voltage/insertion force of the sensor signal after amplification and conversion is ~127 mV/mN), consistent with previous reports^51^. The same tests were performed on 1% and 2% agarose phantoms, and a consistent trend of output or insertion force growth curve was obtained (Fig. 2f, g). These results show that the probe system can ensure high-sensitivity insertion force monitoring when implanted in tissues with different densities. This feature is essential for achieving early warning of injury to various soft tissues under different environments.

### In vivo insertion force monitoring and early warning of vascular tissue injury

Successful bloodsucking by mosquitoes is enabled by several key factors, such as the proper use of the positive pressure of blood in the vessels. We focused on two advantages related to the structure and function of mosquito mouthparts: (1) The specialized multilayer mouthparts enhance the critical buckling load and decrease the insertion load^50^; and (2) the mouthparts exhibit a refined ability to locate blood vessels^48^. By adding the nested track structure and sharpening the shuttle tips, the probe system achieves an implantable performance similar to advantage 1. Mimicking the effect of advantage 2 above was achieved by adding high-sensitivity pressure sensors and, conversely, was used for early warning of vascular damage. In the following in vivo implantation experiments, we used the probe system to penetrate the dura, blood vessels, and basic brain tissue of mice and evaluated the above two abilities according to stress feedback.

As shown in Fig. 2i, the implant module can easily penetrate the dura and deliver four shanks from the flexible electrode array into the brain tissue. After the bioglue between the shuttles and shanks degrades, the shuttle can be stably withdrawn along the track, with minimal surgical trauma. Ten sets of penetration force peaks were calculated when the sharpened shuttle penetrated the dura, and the average value was ~3.8 mN. The typical shuttle needle used in the experiment has a diameter of 40 μm, a cross-sectional area of ~1256 μm^2^, and an approximate average pressure of 3.03 × 10^6^ Pa. These results are similar to previous findings^19,23,52^. We also calculated the insertion force required for the flat tip shuttle of the same diameter to penetrate the dura mater and compared it with that of the experimental group (Fig. S8). The results showed that the unsharpened shuttle obviously required a much higher puncture force, with an average value of ~18.9 mN, which was nearly five times that of the experimental group. Reducing the penetration load by creating a sharp tip has proven to be one of the shortest ways to achieve transdural delivery^32^. This technique minimizes pressure on the brain and avoids the extra time and damage of the dura removal surgery, as well as the depth-targeting error that can result from brain swelling after dura removal.

Then, we conducted the penetration test and insertion force monitoring on intracranial blood vessels and brain tissues of mice, respectively. Before insertion, protective tissue such as the dura on the surface of the mouse brains was carefully removed to avoid interfering with the test results. We selected blood vessels that were clearly visible on the surface of the mouse brain as test subjects. We observed the whole insertion process of the blood vessel synchronously through a high-magnification microscope and used this optical inspection to determine whether the blood vessel was punctured by the bleeding site after the withdrawal of the probe. Figure 2j shows the sensor output curve for representative probe penetration of intracranial vessels and peak statistics for ten penetrations. The average insertion force required to penetrate the vessel is approximately four times higher than that of the basal brain tissue, ~1 mN, which is similar to that reported previously^51–53^. These results indicate that if the probe touches a vessel during implantation, then we can easily obtain an early warning signal based on abnormally high sensor output and avoid further insertion that might rupture the vessel. This tactile (rather than visual) feedback method provides the basis for supplementary closed-loop invasive deep brain surgery and presents broad application prospects in the field of neuroscience and clinical brain diseases.

### Postoperative in vivo records of awake moving mice

**Fig. 3:**
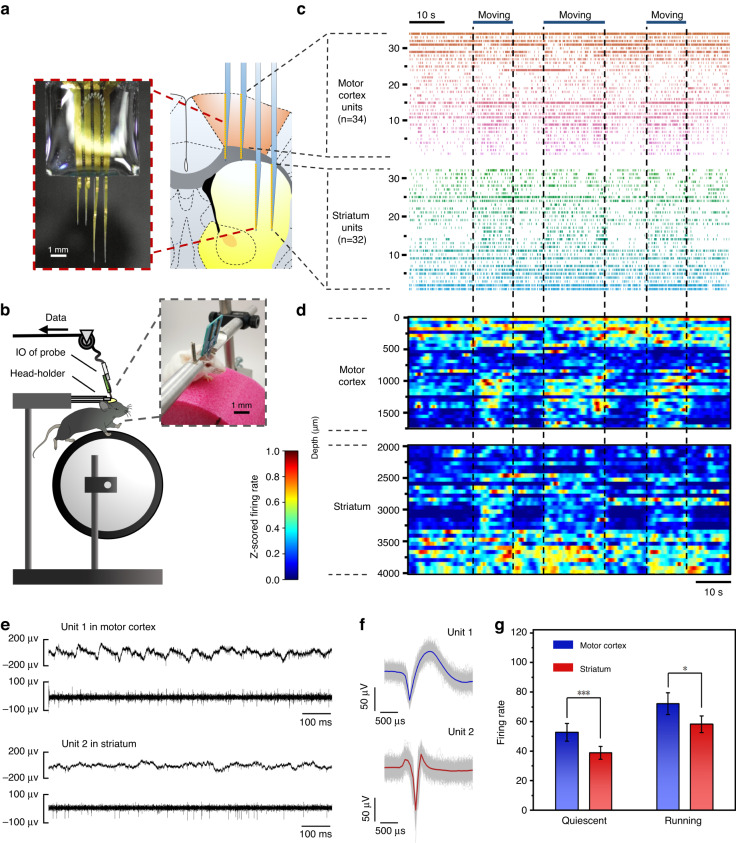
**Postoperative in vivo records of awake moving mice**. **a** A customized implantation display in which flexible electrode arrays were implanted in the motor cortex and striatum of mice. **b** Schematic diagram and photos of the test platform. **c**, **d** Raster map and firing rate heatmap of neural signals collected from the motor cortex and striatum of the mouse 12 h after surgery. **e** Example raw data traces show local field potentials and spiking (above) and high-pass filtered signals (below). **f** Average action potential shape is shown for the recording in **e**. **g** Significantly higher spiking rates were observed during running in both motor cortical and striatal recordings, and the data were collected from the 66 units and a total of 10 experiments

The ability of the invasive electrode device to collect neuronal activity with a high signal-to-noise ratio promptly following implantation is an important metric in determining the invasiveness of both the surgical procedure and the device itself^2,8,54^. In the following in vivo recording experiments, we used the distributed implantation capability of the probe system to evenly implant the flexible electrode arrays into the motor cortex and dorsal striatum of mice (Fig. 3a). Recordings were performed acutely within 12 hours of device implantation, and mice were allowed to run voluntarily on a wheel treadmill in a head-constrained condition while recording (Fig. 3b). Approximately 66 units (34 units from the motor cortex and 32 units from the striatum) were reliably identified across two brain regions during a typical 10-min recording session (Fig. 3c), yielding 0.57 (66/116) single units per channel, which is higher than the results of a previous study^6^. Representative neural electrical activity is illustrated in Fig. 3e, f from the cortex and striatum, respectively. The acquisition of large-scale neural activity within a short time after surgery is strong support for the minimally invasive effect of flexible probe system implantation. Then, we discovered that neuronal spike activity was highly correlated with animal motion in the motor cortex and striatum recordings. We compared the spiking rates of the above 66 units in the running sessions and the quiescent state and collected the data from a total of 10 experiments. The statistics showed that under the running state, the average unit firing rate from the motor cortex and striatum increased by 36.86% and 50.18%, respectively, compared with the quiescent state (Fig. 3g), which are similar data to those found previously^54–57^.

These results indicate that our flexible neural probe system is a convenient tool for ethical studies and large-scale neural activity monitoring across brain regions. In contrast to in vivo calcium ion imaging, which is also used to monitor large amounts of neural activity, the system can record brain regions across different depths^58,59^. This effect is realized by the high degree of customization and freedom of the probe system during implantation. In addition, in vivo imaging often requires virus expression several weeks in advance^58^. The minimally invasive probe system enables the observation of neural activity in a shorter period of time after surgery, which shortens the experimental period and reduces uncertainty during the experimental process. This can prevent failures such as the accidental death of the experimental animal.

### Recording stability and biocompatibility of chronically implanted flexible electrode arrays

**Fig. 4:**
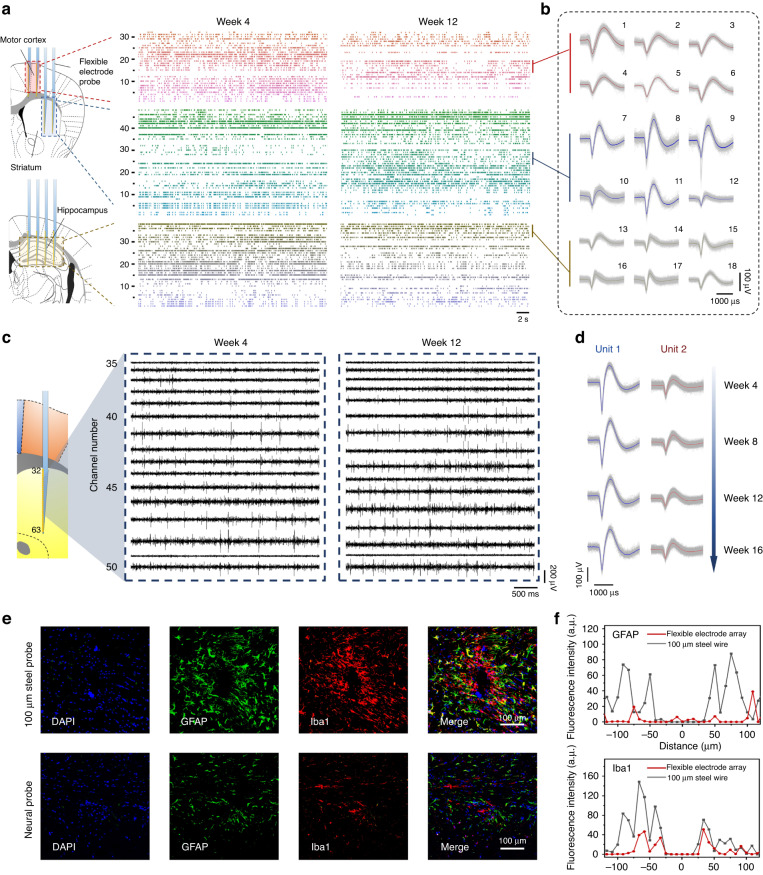
**Recording stability and biocompatibility of chronically implanted flexible electrode array**. **a** Spiking raster of simultaneously recorded neurons by a total of 194 electrodes implanted in the motor cortex, striatum and hippocampus in mice at 4 and 12 weeks postimplantation. Gaps in the raster map represent iterations of neural signals that would occur (left) or had occurred (right). **b** Example spike waveforms from **a**. **c** Representative real-time action potential traces at 4 and 12 weeks postimplantation. **d** Representative time-dependent waveforms of isolated neurons from Ch-41 in **c**. **e** Immunohistochemistry study of different implantable devices. Confocal fluorescence images of 20 µm thick slices for astrocytes (GFAP, green), microglia (Iba1, red), and nuclei (DAPI, blue). **f** The fluorescence intensity of GFAP and Iba1 was plotted against the distance from the implant center of the neural probe and steel probe, respectively

The electrochemically modified electrode interface and mechanical consistency with brain tissue make the flexible electrode array a promising candidate for high-density, long-term recording of neural activity^2^. To assess the long-term stability of the devices, a 128-channel, 2.5-μm-thick electrode array was implanted in different brain regions of wild-type mice (*n* = 3) using the distributed implant function of the probe systems. These mice were observed and recorded for 16 weeks after implantation of the electrode array. The mice were placed in square boxes and allowed to roam freely in the recording process. During this period, most of the recording channels of the electrode array maintained stable impedance, and a large proportion of the neurons recorded by the device obtained stable tracking records. Representative multichannel recordings of neural signals from the motor cortex, striatum, and hippocampus are shown in Fig. 4a. At the fourth week after implantation, we collected 30 (motor cortex), 34 (striatum), and 35 (hippocampus) isolated unit signals from a total of 194 recording channels in three brain regions, which yielded up to 0.51 units per channel. In the 12th week after implantation, we analyzed the signals collected from the same 3 brain regions again and obtained 21 (motor cortex), 44 (striatum), and 30 (hippocampus) isolated unit signals. During the 8-week period, 19 newly recorded unit signals were observed in three brain regions (motor cortex 2, striatum 14, hippocampus 3), while 23 disrupted unit signals were recorded (motor cortex 11, striatum 4, hippocampus 8). This indicates that the flexible electrode array maintained a stable recording yield (total reduction of only ~1 percentage point) and that 76.8 percent of the above 99 unit signals recorded at week 4 were consistently tracked throughout the following eight weeks. Notably, at week 12 after implantation, the unit signals from each brain region still maintained high-quality waveform characteristics and a high signal-to-noise ratio (Fig. 4b). These results indicate that our flexible probe system can be applicable to large-scale, multibrain region, and long time-scale neural recording.

The ability to track the same populations of neurons stably for a long time-scale is an important index for assessment of the biocompatibility and reliability of invasive electrode devices^1^. Compared with the rigid probe, the flexible electrode probe reduces the chronic migration between the recording site and the recorded neuron due to its better mechanical compliance with the brain tissue^6^. In Fig. 4c, we demonstrate representative action potential traces recorded at the 4th and 12th weeks postimplantation from 16 electrode channels implanted in the striatum. It was clear that most of the channels displayed exhibited high signal-to-noise spike waveforms at weeks 4 and 12, indicating that these recording sites remained close to the same neurons throughout the entire period. Principal component analysis (PCA) was performed on representative signals from channel 41, and two unit signals were favorably isolated (*L*_ratio_ = 0.0199 ± 0.0053 and isolation distance = 52.45 ± 18.82) (Fig. S9a). We further analyzed succeeding changes in both units until the end of the 16-week period of recording. During weeks 4–16, the waveforms, amplitude, and corresponding PCA clusters of the two units maintained a high degree of consistency (Figs. 4d and S9a). Further analysis of the interspike interval (ISI) distribution of firing neurons showed that both units exhibited stable firing patterns from 4 to 16 weeks after implantation (Fig. S9c). In addition, we conducted statistics on the firing rates of the two units in weeks 4–16 (Fig. S9b). These results showed that these firing rates were stable over time, with significant differences among different units (*P* < 0.05). The comparison results of PCA prove that the recorded units maintain stable waveform characteristics, and the analysis and results from ISI and firing rate indicate the consistency of discharge activity characteristics. From these data, we infer that the signals recorded by the channel during this period are from the same neurons. The above results prove that our flexible electrode array can realize long-term stable tracking of neuron populations based on its own device performance, rather than relying on post algorithm position correction, which is of great significance for long-term neural circuitry research.

We then evaluated the lesion and immunoreactive responses caused by deep brain implantation of the device. We used stainless steel wire similar in size to the commercial probe (EL30030.01A10, Microprobe, MD) for comparison. Stainless steel wires (100 μm diameter) and flexible electrode arrays were implanted into the mouse brain and sectioned, and immunohistochemical analysis was performed 4 weeks after surgery to quantify scar and microglial cells. As shown in Fig. 4e, the electrode array caused significantly less glial scarring and microglial aggregation than the steel wire As demonstrated by a comparison of average fluorescence intensity curves in Fig. 4f. This improvement is attributed to the extremely small cross-sectional area (the maximum width of the device shank is over 100 μm, but the device thickness is only ~2.5 μm) of the electrode array and the excellent mechanical compliance decreasing tissue-interface displacement and the immunoreactive glial response induced by brain implants^60^. The above results demonstrate that the device has favorable biocompatibility and long-term in vivo working potential. Combined with minimally invasive closed-loop implantation of the flexible probe system and long-term stable recording performance, the system is a promising candidate for brain-machine interfaces and clinical brain disease research.

## Discussion and conclusion

In this study, we present a novel biomimetic neural probe system that incorporates high-sensitivity sensors and a flexible electrode array with 128 channels. This system is designed to emulate the structure and function of a mosquito mouthpart. Our results show that this system offers several key advantages: (1) Customized and distributed implantation of the flexible electrode array is achieved through the use of a configurable implant module. This technique enables the implantation of the electrode array into different brain regions, such as the motor cortex, striatum, and hippocampus of mice, thereby enabling the monitoring of large-scale neural activity across various regions of the brain. (2) The implantation procedure can be performed with minimal surgical intervention and obviates additional dural removal, thereby reducing the potential for intraoperative injury. This benefit is achieved through the use of an improved implant shuttle (with diameter of ~40 μm) with a puncture cross-sectional area of only ~1256 μm^2^ and a sharpened tip that reduces dural penetration force by ~80%. (3) The system provides early warning for potential vascular tissue damage during implantation through haptic feedback, enabling closed-loop implantation surgery. This feature is enabled by a highly sensitive sensor array with a sensitivity of 0.312 mV/kPa^−1^. The highly integrated sensor packaging also allows for miniaturization of the entire system, minimizing the impact on the surgical procedure and reducing the usage barrier. (4) The system demonstrates outstanding postoperative performance, with a unit yield of up to 57% recorded 12 h after surgery. Additionally, the system is capable of stably tracking large-scale neural activity over the long term, with 77% of unit activities being tracked from 4 to 12 weeks after recording began and some unit signals being tracked up to 16 weeks. In conclusion, our study presents a promising direction for the development of multifunctional brain-computer interface devices through the combination of freely customized and minimally invasive implantation, tactile warning, and stable tracking of neural signals.

In this work, we sought to miniaturize the device and to lower the usage barriers, while also improving customization and scalability to better enable additional functionality. For example, by arranging microtubule tracks in three dimensions, it is possible to achieve more customized three-dimensional distributed electrode implantation. By replacing the material of the shuttle and adding a biomimetic microtopographic structure at its tip, the penetration capability of the implanted module is further improved. In addition, this probe system still requires a 3-D stereotaxic instrument to assist implantation. In our future efforts, we plan to further automate the entire system to improve the efficiency of assembly and improve the implantation accuracy using advanced processing techniques. Based on the advancements in functionalities, materials, and processing, we propose that our bioinspired neural-interface system can help realize significant both clinical and research applications in neuroscience.

## Experimental section

### Fabrication of the tactile sensor

The manufacturing process is shown in Fig. S2. After thermal oxidation of the wafer’s front, the first lithography step was performed to locate the piezoresistors. The piezoresistors were formed by boron ion implantation followed by the drive-in process. After low-pressure chemical vapor deposition (LPCVD) of the 0.3 μm thick low stress SiO_2_ layer and 0.8 μm thick TEOS (tetraethyl orthosilicate) layer, the second photolithography was performed to pattern the cavity-releasing microholes with silicon deep reactive ion etching (DRIE). Then, a low stress silicon nitride film with a thickness of 0.2 μm and a TEOS film with a thickness of 0.2 μm were deposited by the LPCVD method. Low-stress silicon nitride and TEOS composite layers deposited at the trench bottom were selectively etched off by RIE to expose bare silicon at the bottom surface of the holes. Then, the silicon DRIE was processed again to deepen the hole to determine the height of the pressure reference cavity. Aqueous TMAH (25%) was used to release the interhole cavity completely by lateral etching at 80 °C. After that, 4 μm thick conformal poly-silicon was deposited by LPCVD to seal the sensor. This was followed by sputtering and patterning a layer of 0.6 μm thin aluminum film to form the interconnect of the piezoresistive Wheatstone bridge. Finally, the shape of the beam-island structure is determined by another RIE.

### Integration of the tactile sensors

The integration process is shown in Fig. S3. Altium Designer software was used to design the flexible printed circuit (the location of the sensors was designed with substrate thickening). Before packaging, the tactile sensors are attached to a flexible printing circuit by vinyl to form an array. Ultrasonic wire bonding is used to lead out the I/O interface to meet the requirements of the compact arrangement of the device. After that, vinyl was applied to the wire bonding area as a protective layer and left on the hot plate at 105 °C for 45 min. Finally, silica gel was applied on the surface of the sensing area of the tactile sensor and solidified for 24 h. After integration, the sensor array was attached to the 3D printing module for further testing.

### Characterization of the tactile sensing arrays

The sensor array is connected to the self-designed data serial bus for power supply and signal preprocessing. The output voltage signal was amplified 44 times by an instrument amplifier array (AD 8221) and filtered by an AD A4522 chip. The amplified signal was collected by a data acquisition card (NI 6255). Digital force measurement equipment was used to apply external forces to the tactile sensor array and test its input‒output response. The LabVIEW program was used to collect data from different channels of the data acquisition card at a sampling rate of 3000 points per second for further experiments.

### Fabrication of microelectrode arrays

We fabricated engineering flexible microelectrode arrays by standard microfabrication techniques. The key fabrication steps are as follows: first, a layer of 2-μm-thick silicon dioxide was thermally grown on the wafer (n-type 0.005 V·cm, XiaMen LuYuan Science and Technology, China), and then a 100-nm-thick nickel was patterned through photolithography and E-beam evaporation as the sacrificial layer to help the device free-standing. After that, a 1.2 μm thick layer of PI (PI-2610, HD Microsystems, USA) was spin-coated on the wafer to form the bottom and top insulating layers. Then, the wafer was cured at 380 °C for 12 h in a nitrogen oven. The interconnects, microelectrodes, and bonding pads of the device were formed by photolithography and metal deposition with a 5-nm-thick chromium layer, a 150-nm-thick nickel layer, and a 50-nm-thick gold layer, respectively. The wafer was subjected to the RIE etching process under the protection of an aluminum hard mask to expose microelectrode sites and bonding pads. After that, the silicon wafer was etched with an aluminum etchant to remove the hard mask protective layer. At the end of the microfabrication processes, the bonding pads of the device were flip chip bonded to a 0.8 mm thick PCB (Shenzhen Jiadubo Electronic Technology, China) through the process of reflow welding, and then the flexible microelectrode array was released from the silicon substrate using the nickel etching agent. The structural design and machining details of microelectrode arrays can be found in previous electrode arrays. Each electrode recording point of the microelectrode array is electrically connected to a corresponding bonding pad on the wafer substrate. We can simultaneously address and record 128 electrode channels through two 64-pin Molex interfaces on the PCB.

### Electrochemical deposition of PEDOT

The deposition of PEDOT on the exposed electrodes of the device was conducted with an electrochemical workstation (CH Instruments, China). The solution of 0.01 M Ethylenedioxythiophene (EDOT, Sigma‒Aldrich, USA) and 0.1 M sodium dodecyl-benzene sulfonate (Sigma‒Aldrich, USA) was prepared in a mixture of 20/80 vol% isopropyl alcohol and water. The microelectrode array is connected to the working electrode position, and the reference electrode (Ag/AgCl electrode) and the counter electrode are connected to a platinum wire in contact with the EDOT solution. Electrochemical deposition was performed in potentiostatic mode at 0.8 V at room temperature. The microelectric electrode array was then cleaned with deionized water and soaked for 24 h before drying for use.

### Assembly of implant modules

We used 3d modeling software (SolidWorks 2014) for the design of the necessary accessories of the implant module. The base of the microtubule track module was manufactured by two-photon 3D printing. The microtubules (polytetrafluoroethylene tubes, O.D. 200 µm, I. D. 100 µm) were prealigned through grooves in the base and fixed in position by epoxy resin. Then, the ends of the microtubule were cut to a specified length. The thinning and sharpening of tungsten shuttles were accomplished by electrochemical etching in 0.8 M NaOH solution (Sigma‒Aldrich, USA). The 75 µm diameter tungsten wires were connected to the working electrode position, and the counter electrode was connected to a platinum wire in contact with the NaOH solution. A constant voltage of 2.5 V was applied to the working electrode for ~1 min to reduce the diameter of the tungsten wires to 40 µm. After whittling the tungsten wire to the specified diameter, a sharp tip is formed at the end of the tungsten wire by positioning its tip ~2 mm below the solution level and etching it at the same voltage for an additional 3 min. The tungsten wire was then threaded through the microtubule track under microscopic view, and the unetched end of the tungsten wire was trimmed to a specified length. After that, the unetched end of the shuttle is wrapped in a 100 μm thick polyimide layer and flattened to form a contact surface with the sensor. Then, the microtubule track module and the prefabricated 3D-printed parts were bonded to the silicon substrate of the microelectrode array using polyethylene glycol (PEG) solution. The 3D-printed parts are designed with clasps for prealignment with other parts (Fig. S4a). Under the microscope, a PEG solution (30,000 molecular weight, Sigma‒Aldrich, USA) was used as a biological binder to fix the four shanks of the flexible electrode array radially to the corresponding shuttle and align the tips. The device was then placed in a fume hood to dry for 3 h in preparation for implantation.

### Implantation of the device into the mouse brain

All animal procedures were carried out at Shanghai Laboratory Animal Research Center, Shanghai, China. All animal-related experiments were approved by the Institutional Animal Care and Use Committee of Fudan University (approval number: 2019-07-HSYY-SZF-01). Male adult C57BL/6 mice (8 weeks old) were used for these studies. The mice were placed in a rodent stereotaxic frame, immobilized using ear bars, and anesthetized using isoflurane (3% for induction and maintained at 1–2%) in medical-grade oxygen. The skull of mice was exposed and prepared by scalping the crown and removing the fascia with the tip of a scalpel blade. A 1.5 mm × 2.5 mm rectangular craniotomy was performed with a surgical drill on the skull above the target brain region. The micromanipulator was then set up to insert the device’s probe into the mouse’s target brain region at a constant rate of 0.5 mm/s until the probe reached the target position and depth. During implantation, data from the tactile sensor array were recorded, and the real-time mechanical/voltage output curve during the implantation process can be displayed through the interface of the LabVIEW program. If the output of the sensor increases significantly and dramatically, we can determine that the probe has touched a potential blood vessel. Then, we choose to retract the entire probe and reselect the insertion position in the adjacent area. After the implantation, wait for ~5 min for the dissolution of PEG. The sensor module on the upper end of the shuttles was removed by a rotating shaft, and the shuttles were withdrawn along the microtubule tracks. The device was then fixed to the skull with dental cement, and all remaining exposed areas of the flexible microelectrode array were covered to prevent scratching. After the flexible microelectrode array was implanted, vulnerable components such as sensors and 3D printing modules were separated and recovered, avoiding scratching damage from the mouse limbs. The surfaces of the implanted modules and 3D-printed parts were rinsed with PBS solution to dissolve the PEG, allowing them to be separated from the silicon substrate of the device and retracted.

### Electrical recording of neural activity

Voltage signals from the microelectrode arrays were amplified and digitized using the RHD Recording System (Intan Technologies, USA), and a 200 μm diameter stainless steel wire was inserted into the contralateral brain region in mice as the grounding reference. The neural signals are transmitted through a data line, and a head stage of the recording system interconnects with the PCB of the microelectrode array through two 64-pin Molex interfaces (Molex, USA). The sampling rate of the recording system was 30 kHz. A 50 Hz low-pass filter is usually used to separate the LFP signal from the original signal; the spike signal is separated by 300 Hz high-pass filtering. Spike detection and spike sorting were carried out by an Offline Sorter (Plexon, USA).

### Immunohistochemistry and imaging

Four weeks after device implantation, the mice were anesthetized with pentobarbital sodium and intracardially perfused with 4% paraformaldehyde (Sigma‒Aldrich, USA) in PBS solution. The brain was carefully removed and cryoprotected in a 4% paraformaldehyde solution overnight and then sectioned into 20 μm thick slices perpendicular to the probes. Brain slices were incubated in blocking buffer (PBS containing 0.5% Triton X-100 and 3% bovine serum albumin) for 50 min at room temperature. Then, brain slices were incubated for 12 h at 4 °C in blocking buffer that contained rabbit anti-Iba1 (ab178846, Abcam, USA, 1:200 dilution) and mouse anti-GFAP (60190-1-lg, Proteintech, USA, 1:500 dilution). After that, slices were washed three times in PBS and incubated for 50 min at room temperature in blocking buffer that contained Alexa Fluor 488 goat anti-rabbit IgG (bs-0295G-AF488, Bioss, USA, 1:200 dilution) and Alexa Fluor 555 goat anti-mouse IgG (bs-0296G-AF555, Bioss, USA 1:200 dilution). Then, slices were washed three times in PBS and incubated with 4′,6-diamidino-2-phenylindole (DAPI, AR1176, Boster Biological Technology, USA) in 0.1 M PBS that also contained 3% bovine serum albumin for 50 min at room temperature. After being washed three times in PBS, mouse brain slices were mounted on glass slides with 50% glycerin. All slices were imaged using fluorescence microscopy (Olympus, Japan).

## Supplementary information

Supporting Information
