# Supplementary material for: A mosquito mouthpart-like bionic neural probe
Source: Microsyst Nanoeng. 2023 Jul 12;9:88. doi: 10.1038/s41378-023-00565-5 (PMC10336119; doi:10.1038/s41378-023-00565-5)
Supplement: Supplementary file 1 — Supporting Information [file 41378_2023_565_MOESM1_ESM.docx]

Supporting Information

**A mosquito mouthpart-like bionic neural probe**

*Yu Zhou, Huiran Yang, Xueying Wang, Heng Yang, Ke Sun, Zhitao Zhou, Liuyang Sun, Jianlong Zhao, Tiger H. Tao^*^, and Xiaoling Wei^*^*


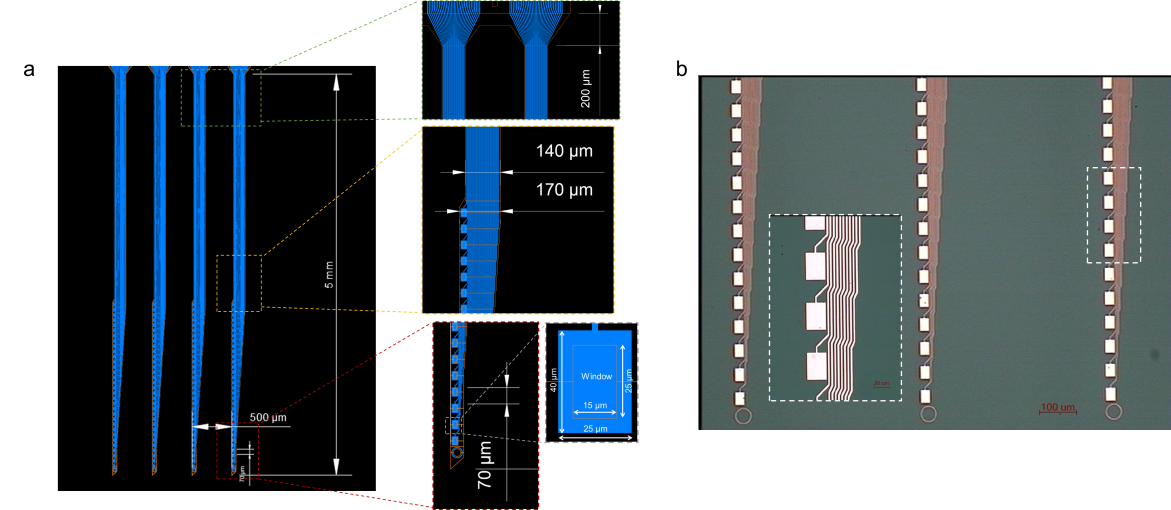


**Figure S1**. (a) Device design of the electrode arrays and magnified views. The microelectrode exposed through the window is 25 × 15 μm in size. The blue section was patterned by a Au layer as interconnects and microelectrodes, while the red section was patterned by PI. (b) Microscopic view of a device (layer of interconnects and microelectrodes) on a wafer.


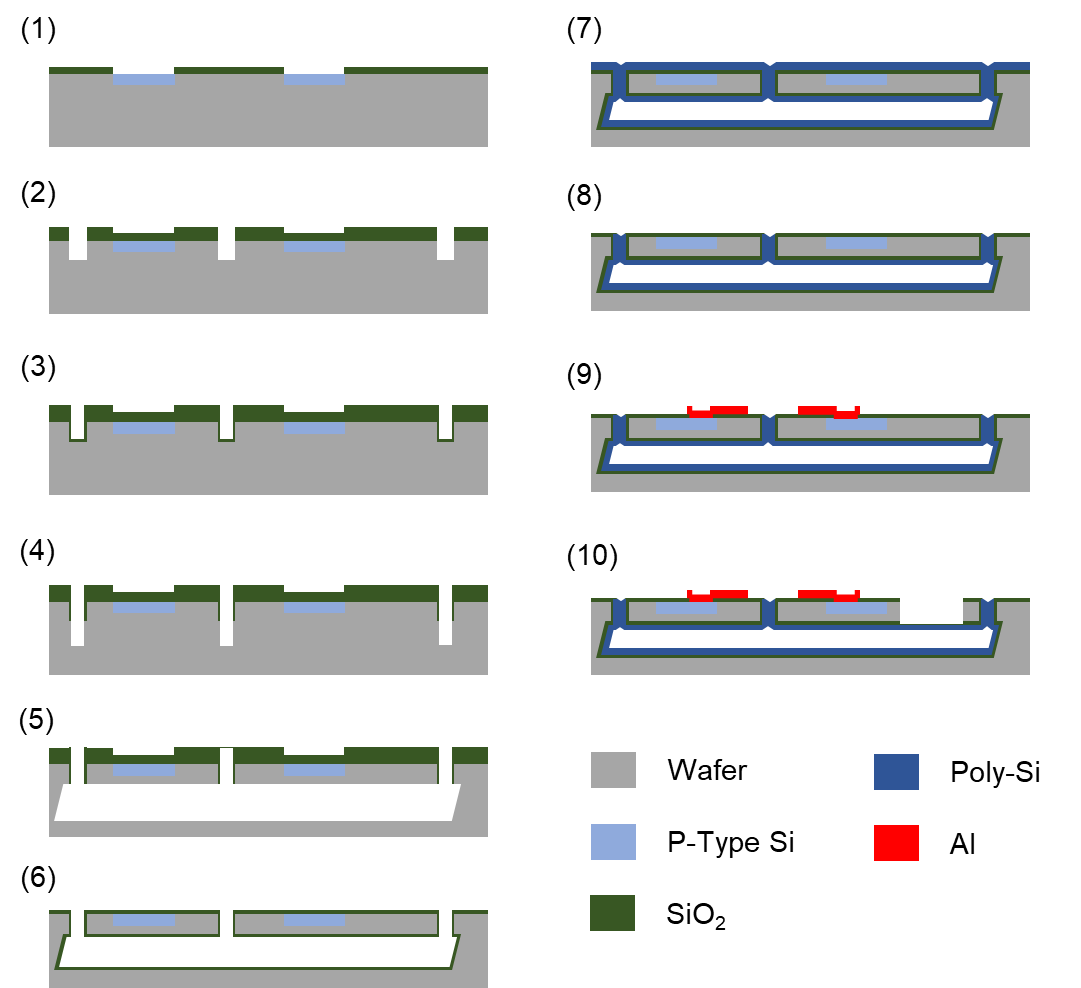


**Figure S2**. Cross-sectional views of the process steps for fabricating the tactile sensor.


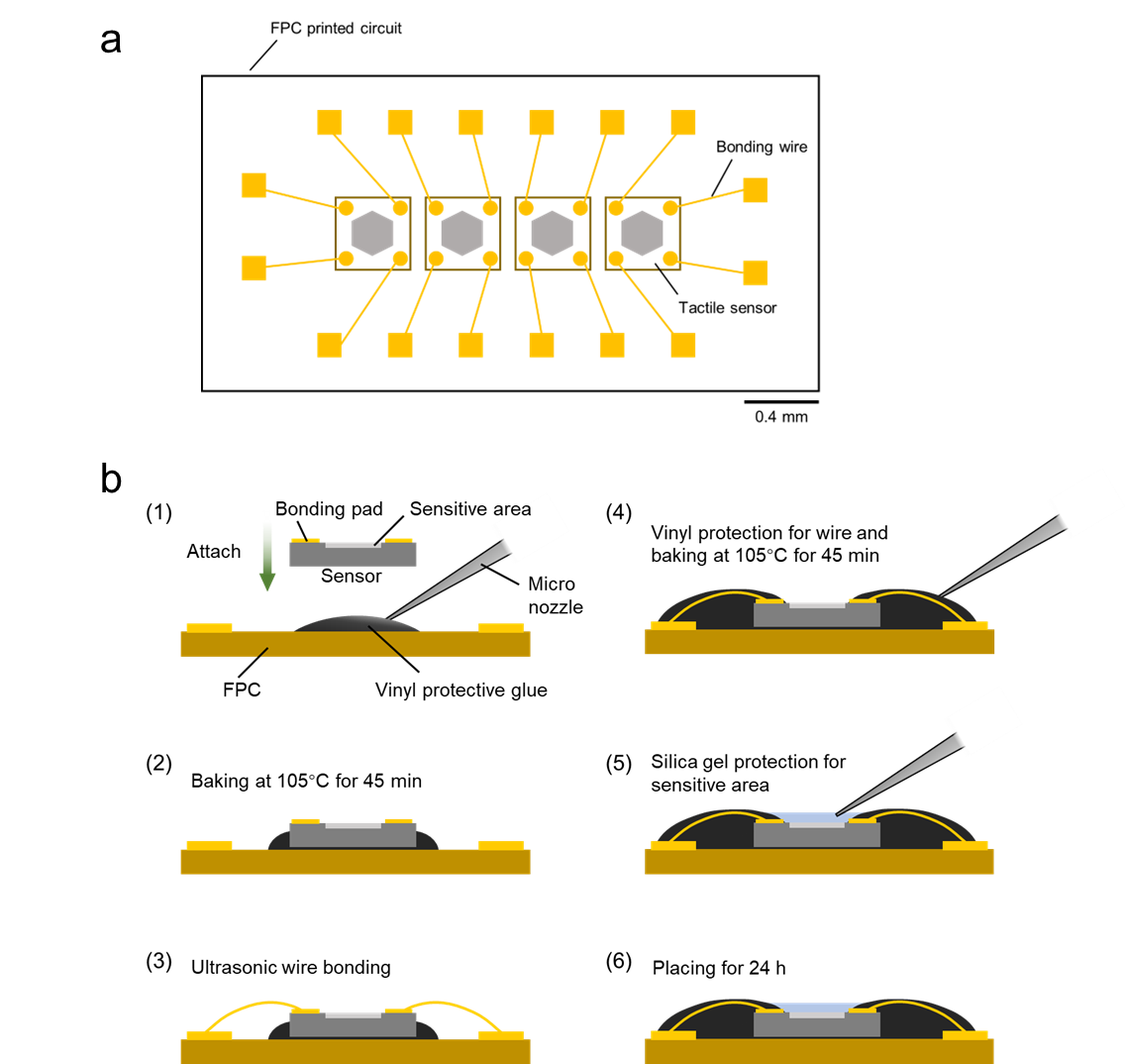


**Figure S3**. Integration of the tactile sensors: (a) Sensor arrangement and position on the FPC printed circuit; (b) Sensor packaging process.


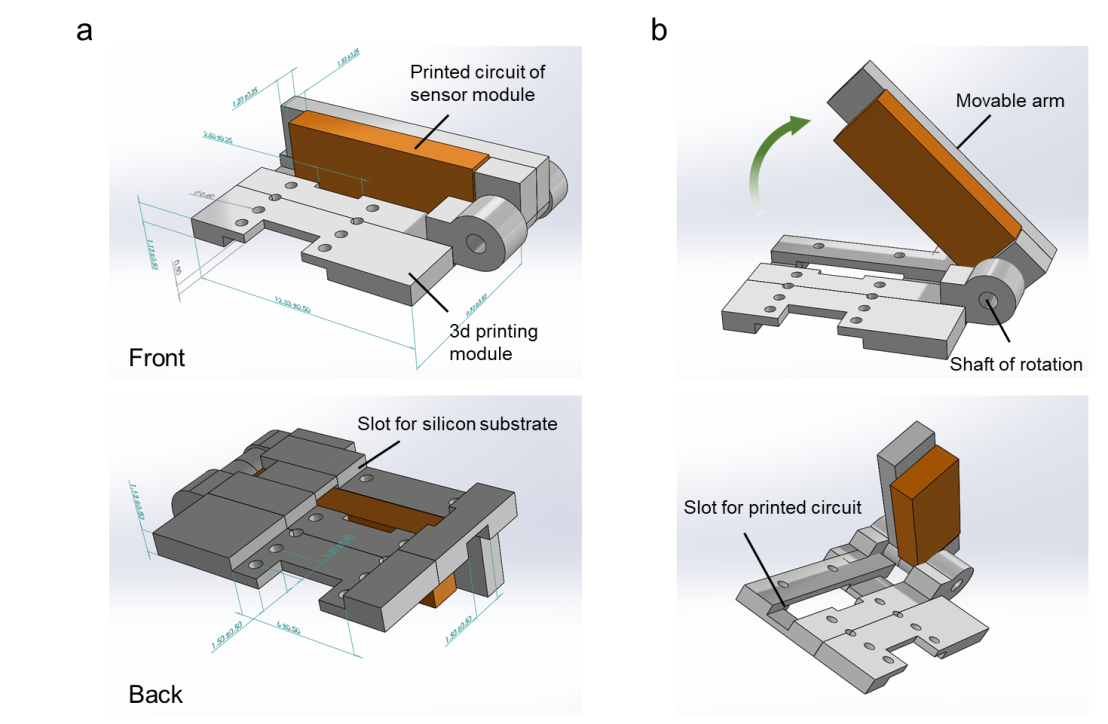


**Figure S4**. (a) Size and structure drawing of the 3d printing module, and (b) movable structural display.


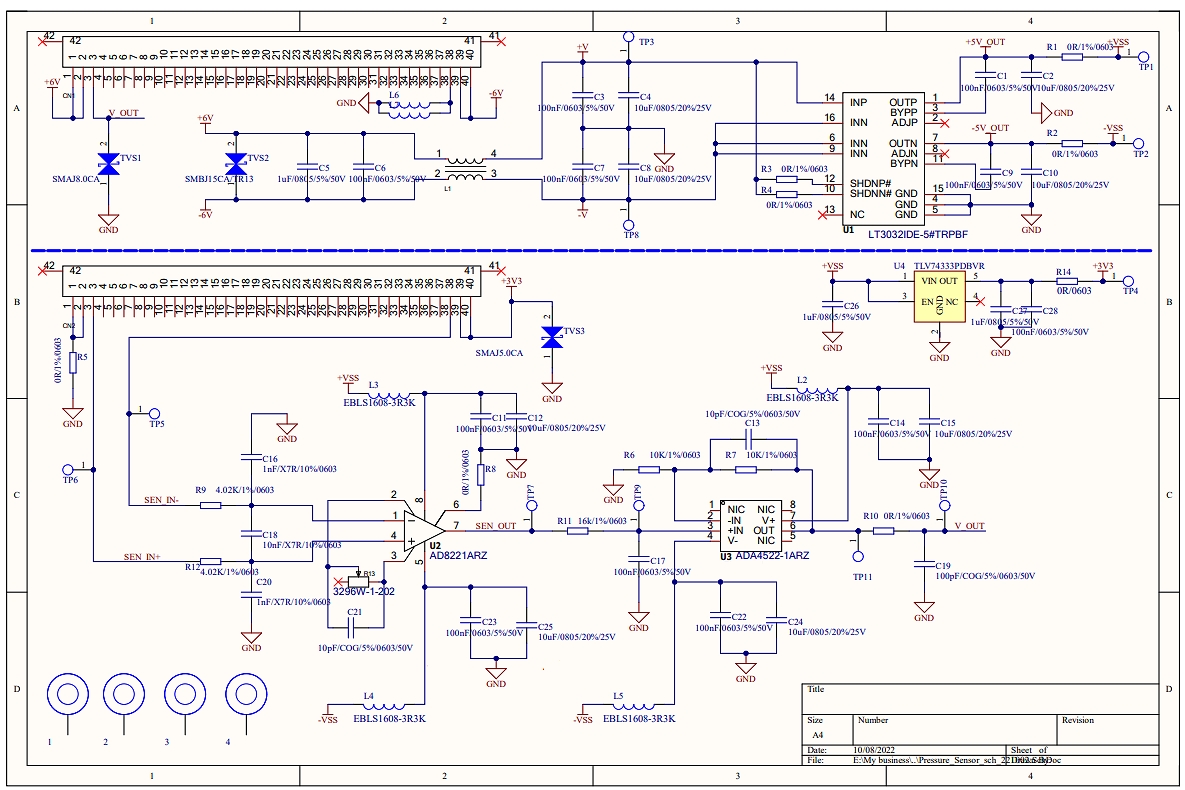


**Figure S5**. Signal amplifying/filtering circuit of the tactile sensor module.


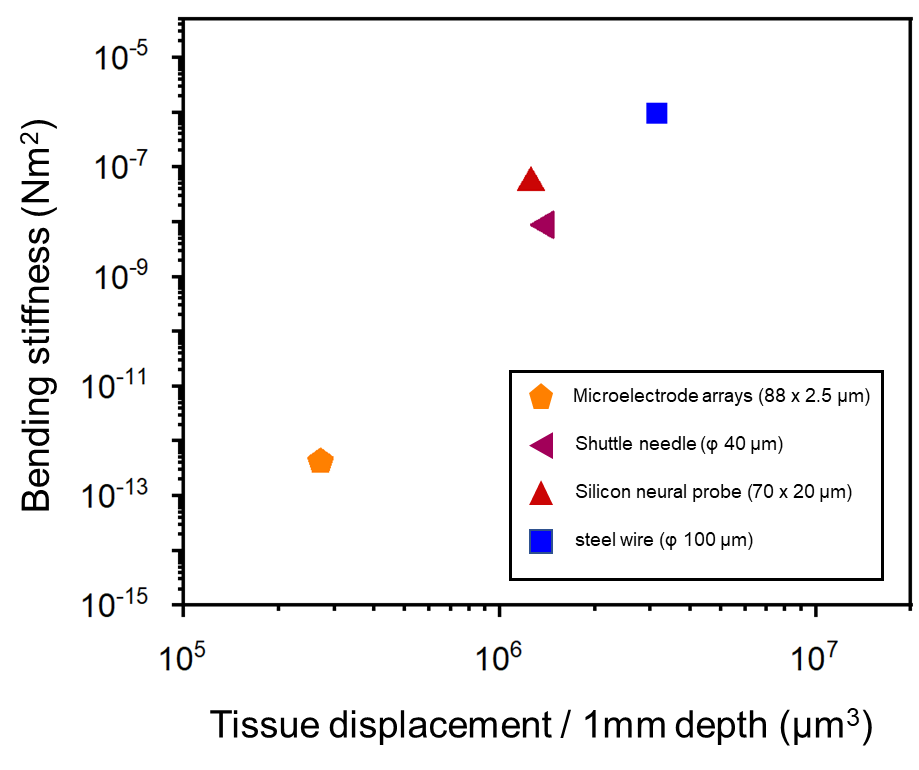


**Figure S6**. Drastically reduced bending stiffness and tissue displacement of the microelectrode arrays compared with other representative probes. For shaft-shaped probes, Ks=E_s_wh^3^/12, where E_s_ is the Young’s modulus of the shaft material and h and w are the thickness and width of the shaft, respectively. For cylindrical probes, Kw=E_w_πd^4^/64, where E_w_ is the Young’s modulus of the wire/fiber material and d is the diameter of the wire. TD/1 mm equals the average cross-sectional area multiplied by the implanted length of 1 mm.


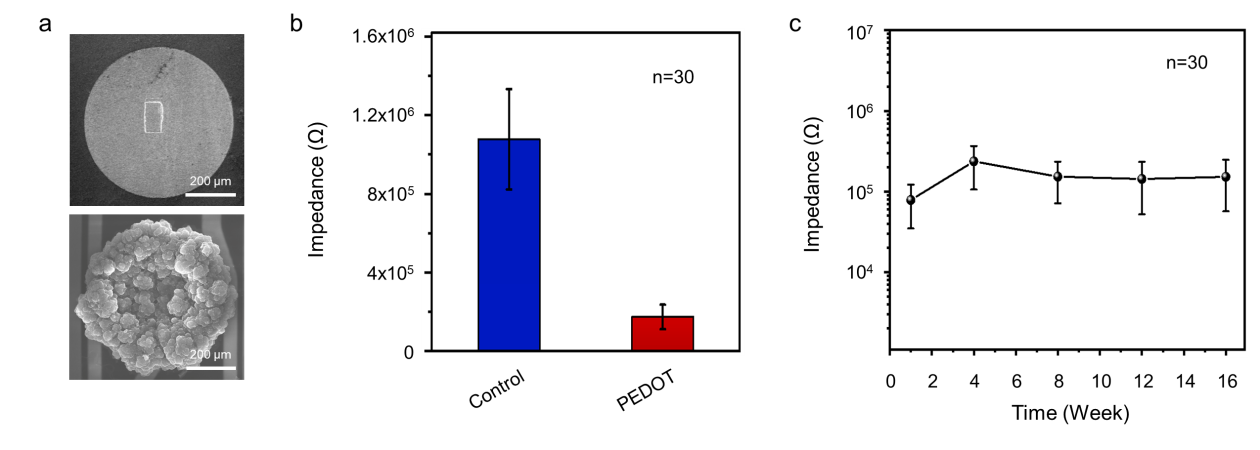


**Figure S7.** (a) SEM images of a typical gold microelectrode before and after PEDOT modification. (b) Impedance statistics of 30 typical microelectrodes before and after PEDOT modification. (c) Impedance changes of the typical 30 microelectrodes during the 16 weeks after implantation.


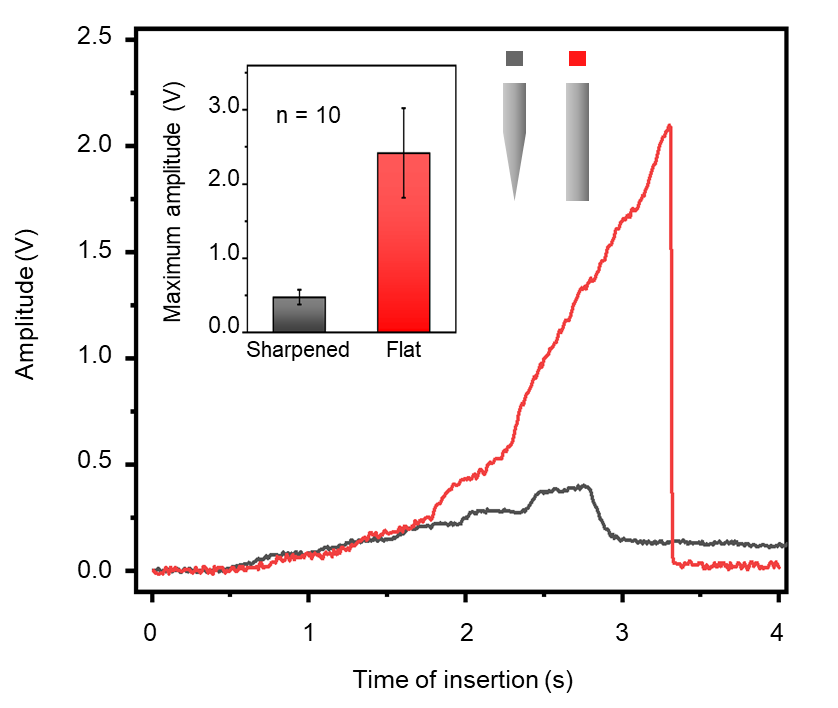


**Figure S8**. Representative output voltage of the sensor module when the probe penetrates the dura of the mouse using the flat tip and sharpened shuttles and the average peak value (embedded picture).


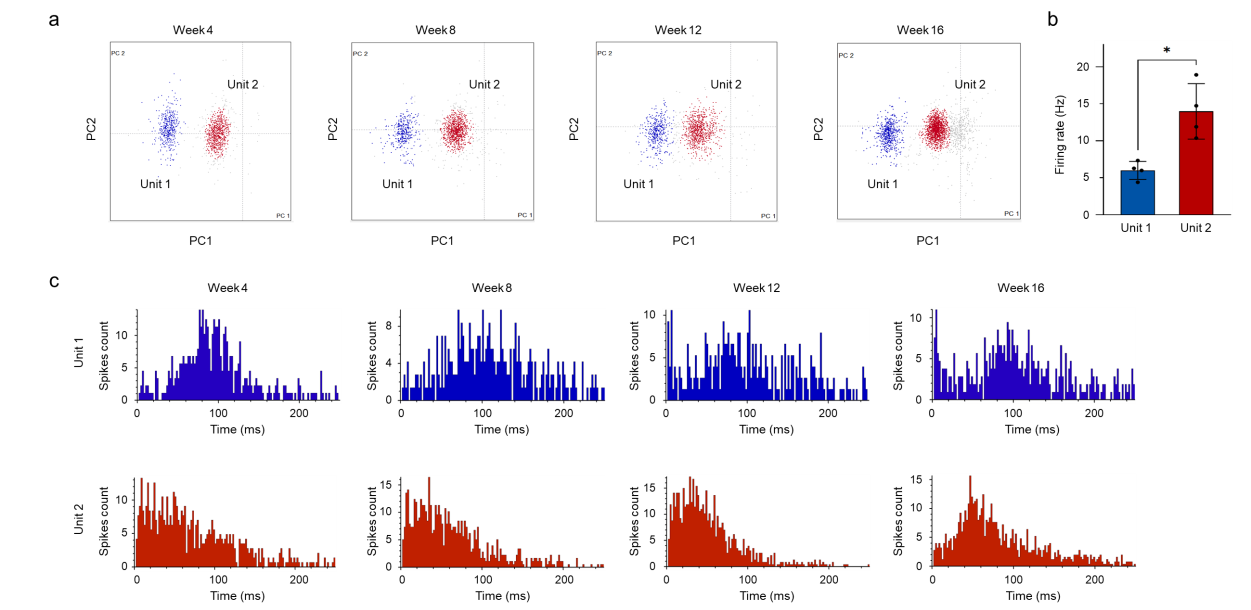


**Figure S9**. (a) Clusters revealed by PCA of isolated unit 1 and unit 2 action potentials in Figure 4d. (b) Average firing frequencies for neurons 1 and 2. Error bars represent s. d. (c) ISI histograms for isolated neurons 1 and 2 from (a).
